# Clinical factors affecting breath‐hold performance for left‐sided breast cancer patients

**DOI:** 10.1002/acm2.70364

**Published:** 2025-11-25

**Authors:** Kaelyn Becker, Kelly Kisling, Laura Padilla

**Affiliations:** ^1^ Department of Radiation Medicine & Applied Sciences UC San Diego Health La Jolla California USA

**Keywords:** breast, breath hold, DIBH, surface imaging

## Abstract

**Purpose:**

Deep inspiration breath hold (DIBH) is a treatment technique used for patients with left‐sided breast cancer to move the heart further from the treatment area and thus reduce cardiac toxicity. Active participation of the patient plays a vital role in the success and efficiency of DIBH. The purpose of this study was to investigate various clinical factors that can influence breath‐hold performance.

**Methods:**

Surface‐guided radiotherapy (SGRT) data from 72 left‐sided DIBH breast cancer patients monitored with AlignRT (VisionRT) were retrospectively analyzed to evaluate breath‐hold accuracy (deviation of patient surface at treatment from the CT simulation surface), reproducibility, and the percentage of breath holds within tolerance (%WT). An internal Python‐based tool was developed to identify all breath holds from acquired breathing traces during patient setup and treatment. Patients were grouped based on clinical factors such as age, English proficiency, and reported pain or anxiety. Group differences in the breath‐hold performance metrics were assessed using the Mann–Whitney *U* test.

**Results:**

Breath‐hold accuracy did not show statistically significant differences across any patient groups. However, reproducibility was significantly worse for patients reporting pain (standard deviation = 2.54 mm vs. 1.69 mm, *p* = 0.0003), indicating greater difficulty in maintaining consistent breath‐hold levels. In terms of %WT, patients with reported anxiety demonstrated lower performance (83.3% vs. 90.2%, *p* = 0.03), as did patients reporting pain (80.6% vs. 86.9%, *p* = 0.02). These findings suggest that both pain and anxiety are factors that may negatively impact a patient's ability to consistently hold their breath during treatment.

**Conclusions:**

This study highlights the importance of addressing patient‐reported pain and anxiety in achieving optimal breath‐hold performance during DIBH treatments. While factors such as age and English proficiency did not significantly impact breath‐hold metrics, pain and anxiety were associated with impaired reproducibility and reduced time within tolerance. These results suggest that targeted interventions, such as pain management strategies, additional coaching, or supportive devices to increase patient comfort, may improve patient outcomes and clinical efficiency for anxious patients or those in pain. DIBH remains a feasible and effective technique for patients regardless of age or native language in the patient cohort we investigated.

## 1 INTRODUCTION

Radiation following breast‐conserving surgery or mastectomy can reduce the risk of local recurrence at the irradiated site by up to 70%.^1^, ^2^, ^3^ While this reduction decreases overall mortality, postoperative radiation is also associated with a 30% increase in cardiac‐related mortality and has been linked to higher rates of chest pain, coronary artery disease, and myocardial infarction.^4^, ^5^, ^6^, ^7^ For each 1 Gy increase in mean heart dose, the relative risk for ischemic heart disease increases by 7.4%.^8^ Therefore, it is important to use treatment techniques that reduce the mean heart dose in breast cancer patients, thereby lowering their risk of heart disease and cardiac mortality.

Deep inspiration breath hold (DIBH) is a radiation therapy technique aimed at increasing the distance between the heart and the treatment field in patients with left‐sided breast cancer. When performed correctly, DIBH significantly lowers the mean heart dose while preserving effective coverage of the chest wall or breast.^9^, ^10^ The SAVE‐HEART study^11^ demonstrated that using DIBH in left‐sided breast cancer patients results in a 5% relative reduction in the 10‐year risk of cardiovascular disease compared to radiation therapy without DIBH. Successful implementation of DIBH requires active patient participation, as patients must repeatedly take a deep breath and maintain the breath hold during treatment.

Several methods exist to monitor or control the patient's breathing during treatment. While active breathing control (ABC) tightly controls patient breath holds by employing a device that suspends a patient's breathing at a reproducible level, this is a technique that can result in patient discomfort. Additionally, even though the tidal volume is the same for each breath hold using an ABC device, the internal anatomy may differ if patients' breathing technique varies (e.g., abdominal vs. thoracic breathing).^12^ An alternative to active breathing control is monitoring the patient's surface in real time using surface‐guided radiation therapy (SGRT), external markers, or portal imaging.^13^ However, SGRT relies on an external surrogate and does not directly verify internal anatomy. Patients may adopt postures that meet surface tolerance yet alter internal anatomy. For example, arching the back can bring the surface into tolerance without preserving the heart‐to‐target distance so careful coaching and monitoring are necessary with SGRT.

At our institution, DIBH radiation therapy is treated using SGRT. This technology tracks the patient's surface in real‐time, comparing it to a reference surface captured during CT simulation.^14^ Predefined tolerances are established such that treatment is paused by therapists if real‐time deviations exceed set limits. By providing continuous, non‐invasive monitoring of breath‐hold alignment without introducing additional ionizing radiation, SGRT enhances the precision and safety of DIBH treatments.^15^

While SGRT effectively monitors patient breath holds to ensure the treatment is delivered only when the patient's breath hold is at the correct level, challenges with a patient's ability to consistently perform breath holds can unexpectedly prolong treatment times. These delays disrupt clinical workflow and may increase patient discomfort and anxiety. The goal of this study is to identify clinical factors that may predict which patients are likely to encounter challenges prior to treatment. Early recognition of these factors will enable the development of targeted interventions to proactively address potential barriers, ensuring that no patient group is excluded from DIBH. To achieve this, we evaluated breath‐hold performance of left‐sided breast cancer patients treated with DIBH at our institution, examining how performance varied with four clinical factors: patient age, English proficiency, and reported levels of anxiety and pain.

## 2 MATERIALS AND METHODS

We retrospectively analyzed all patients with left‐sided breast cancer who were treated using DIBH guided by real‐time surface imaging with AlignRT (v7.0, VisionRT, London, UK) between August 1, 2023, and November 1, 2024 (*n* = 72). During CT simulation, all patients underwent both free‐breathing (FB) and DIBH CT scans, acquired with 2.5 mm slice thickness and extending from the neck to approximately 4–10 cm below the breast. Breath holds were monitored at the time of simulation using SimRT (VisionRT). Patients were asked to perform a breath hold and demonstrate that they could hold their breath at a consistent level for at least 15 s and could perform at least one additional breath hold to the same level before they were considered DIBH candidates. The free breathing and breath‐hold scans were used to generate reference body contours, which served as the basis for surface guidance during patient setup and treatment monitoring.

**FIGURE 1:**
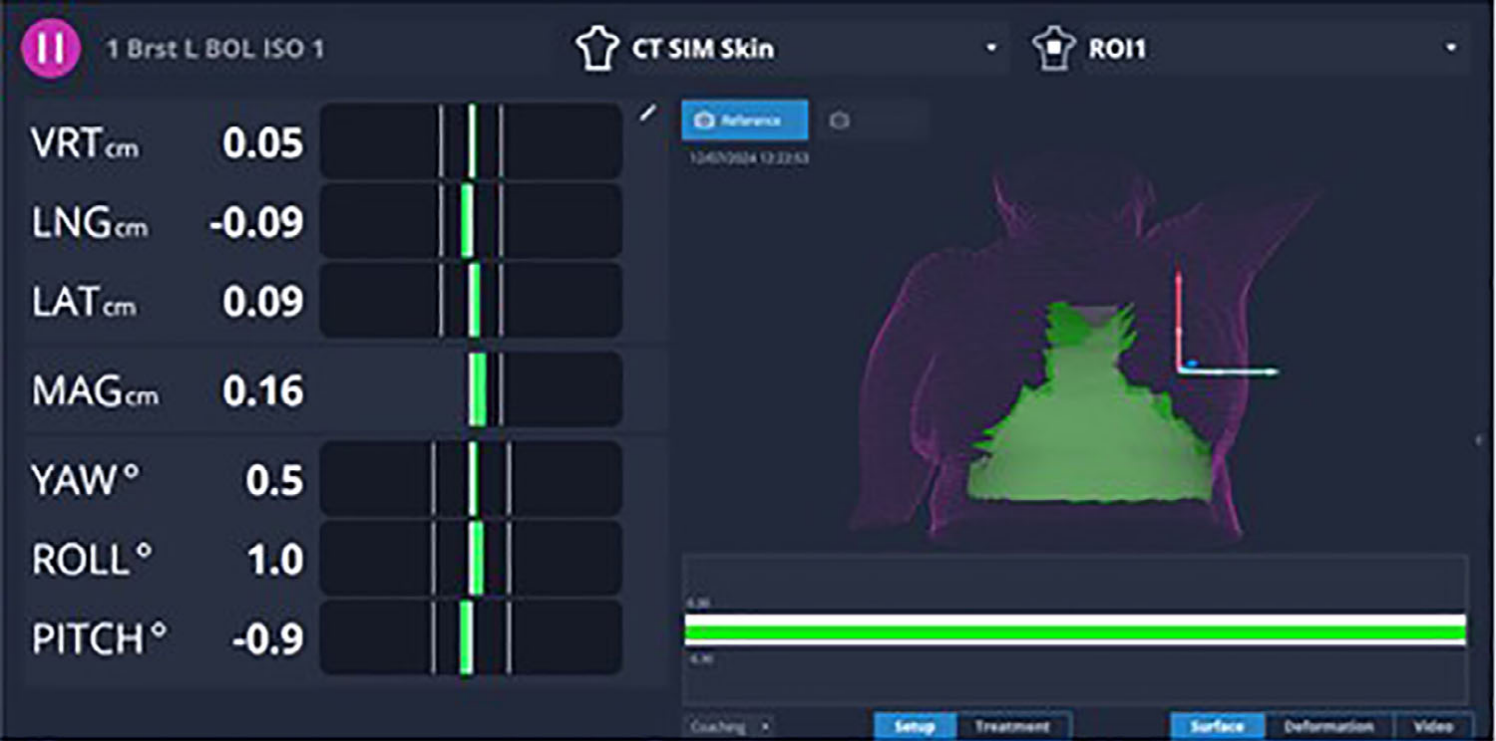
AlignRT user interface for tracking patient breathing during treatment. An “inverted T” region of interest shape was used for tracking.

To ensure accurate breath holds on treatment, an “inverted T” region of interest (ROI) on the reference surface was typically used (Figure 1). This ROI minimizes sensitivity to daily changes in breast volume while capturing lateral regions critical for detecting roll and pitch setup deviations, which can affect the position of the heart relative to the treatment field.^16^ Adjustments to the ROI were made when clinically indicated, for example, to accommodate reproducible abdominal (“belly”) breathing that achieved adequate cardiac displacement. When Superflab bolus was used, a new reference surface that included the bolus was captured. The original DIBH surface was first used to verify breath‐hold position. With the patient maintaining the breath hold throughout, the bolus was placed, and a new reference surface was captured under breath hold. This updated surface was used for monitoring for the remainder of the session. At the beginning of each fraction, the process was repeated.

For this study, we extracted vertical displacement (delta) values from AlignRT log files, comparing the monitored surface during treatment to the planning CT reference within the defined ROI. Institutional Review Board (IRB) approval was obtained for this retrospective analysis.

### 2.1 Patient selection and clinical factors

Table 1 summarizes the clinical and demographic characteristics of the study cohort. Patient‐reported pain was extracted from physician consult documentation, where patients were routinely asked to rate their pain on a scale from 0 to 10. Responses were grouped into two categories: no pain (score of 0) or any reported pain (score ≥ 1), with observed scores ranging from 0 to 7. Anxiety was assessed from the same documentation and categorized based on whether the patient mentioned experiencing anxiety. English proficiency was classified as high (no translator required) or low (translator required), and age was grouped as <65 years or ≥65 years, with a mean of 59.1 years (range: 28–90).

**TABLE 1:** Patient characteristics for all patients included in this study (*n* = 72).

Patient characteristic	Value
Age	Under 65: 46; over 65: 26
English proficiency	High: 64; low: 8
Reported pain	Yes: 19; no: 53
Reported anxiety	Yes: 12; no: 60
Technique	3‐Field: 43; tangents: 29
Bolus	Yes: 18; no: 54

### 2.2 Breath‐hold identification

The user interface of the AlignRT software monitoring the breath‐hold level of a DIBH patient is shown in Figure 1. The differences between the AlignRT surface and reference surface are saved in a log file that can be accessed after each treatment. This data includes breath holds performed during initial patient setup, imaging, and treatment, since therapists use SGRT throughout the whole process.

We developed an in‐house Python (v3.13) tool to extract data from SGRT (AlignRT) log files and identify all breath holds during treatment sessions. The algorithm detects potential breath‐hold periods by analyzing vertical displacement (vertical deltas) between the reference and real‐time surfaces sampled every 100–200 ms. The body contour from the DIBH planning CT was the reference surface for most patients, except for those whose surface was recaptured after bolus placement. In those cases, this recaptured surface was used as the reference surface.

**FIGURE 2:**
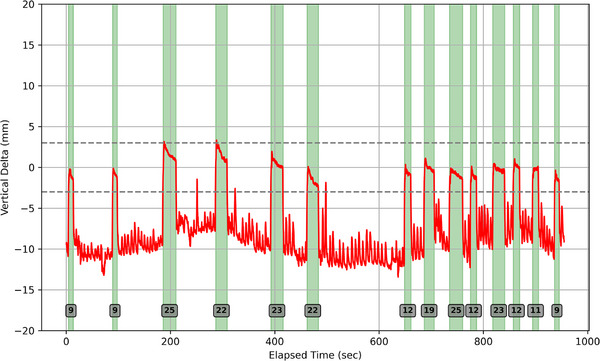
A vertical delta trace from a representative patient. The green highlighted regions mark the start and end of each breath hold and the numbers in the gray boxes show the length of each breath hold. Dashed gray lines show the tolerance limits for treatment (±3 mm).

Candidate breath‐hold regions were identified as segments of the breathing trace that exhibit low variance relative to adjacent time intervals, combined with sustained amplitude plateaus lasting at least 2 s. This approach leverages the physiological differences between free‐breathing and breath‐hold states: free breathing exhibits high surface motion variance, whereas breath‐holds result in consistent surface positioning and larger amplitude. By applying these criteria, the algorithm automatically detects breath‐hold periods during their treatment. Figure 2 shows a representative vertical delta trace highlighting the breath holds identified and the tolerances for treatment (±3 mm).

The model was validated by overlaying detected breath holds on the raw AlignRT traces and manually reviewing each interval across all fractions. The algorithm was intentionally tuned for high sensitivity so that no true breath holds were missed. If the breathing trace was noisy, the algorithm would occasionally over select and highlight a region that was not a true breath hold (precision = 96.8%). After detection, these false positives were manually removed, and all metrics were computed only on validated breath holds.

### 2.3 Breath‐hold analysis

**FIGURE 3:**
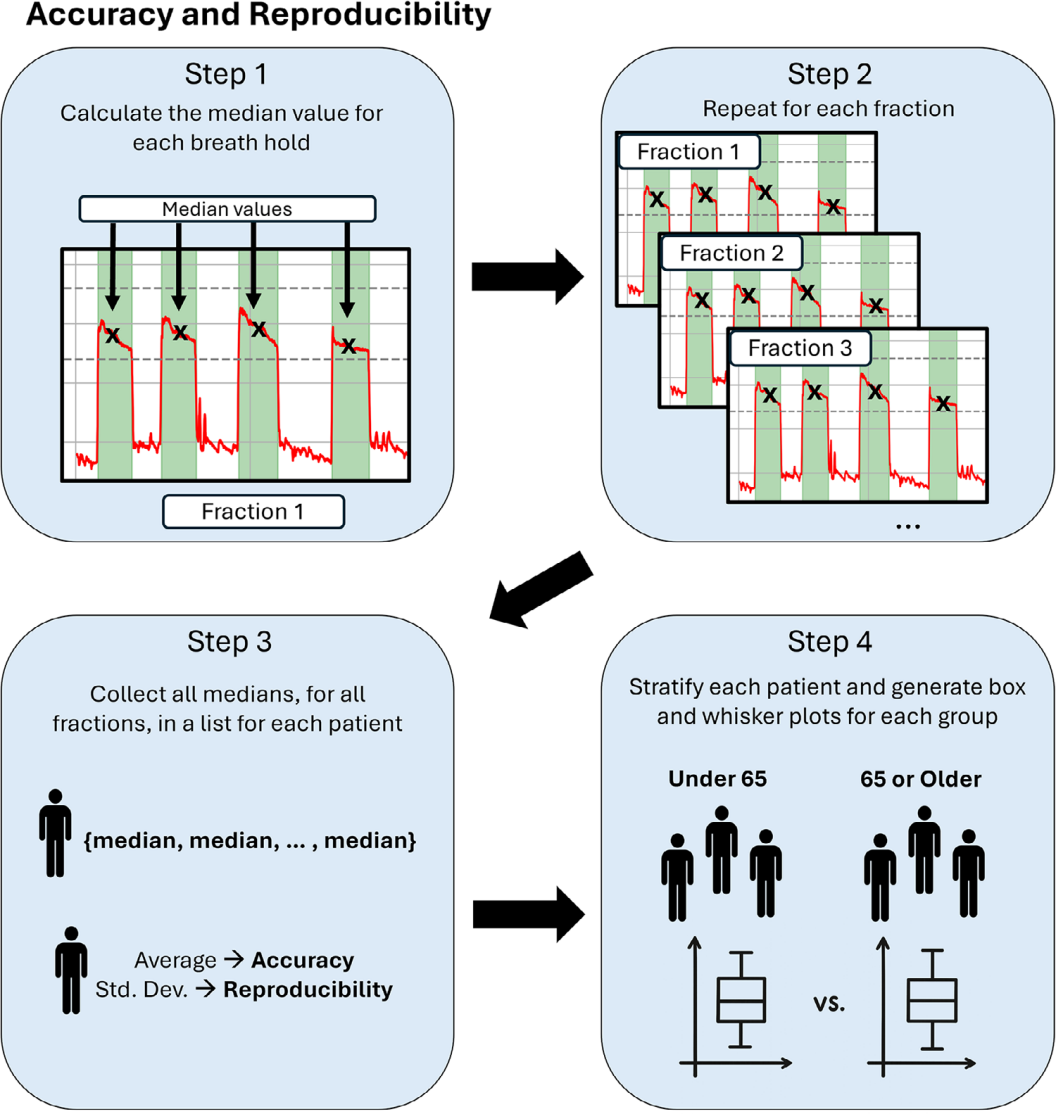
Workflow for determining accuracy and reproducibility.

**FIGURE 4:**
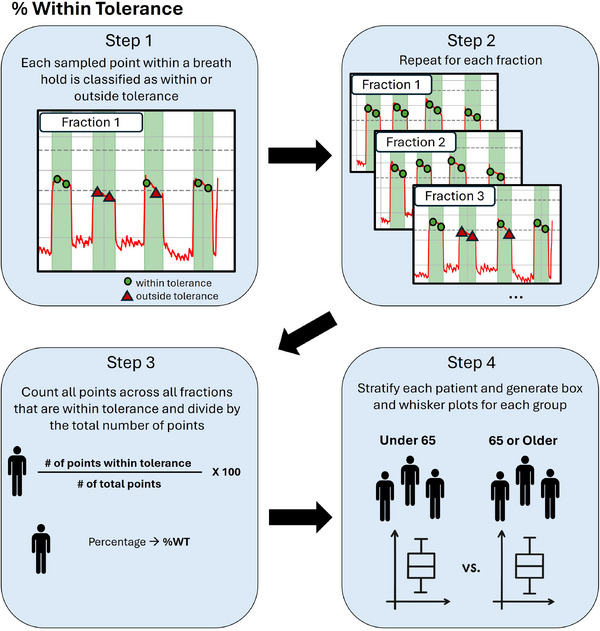
Workflow for determining %WT.

To evaluate breath‐hold performance, we analyzed three metrics from vertical delta values: accuracy, reproducibility, and the percentage of sampled breath‐hold points within tolerance (%WT). Vertical delta values were extracted for each fraction across each patient's course using AlignRT. All metrics were calculated from breath holds regardless of beam status. For accuracy and reproducibility, each breath hold was represented by the median amplitude of the plateau region (Figure 3). Accuracy was defined as the mean of these breath‐hold medians (a mean signed error/bias relative to CT simulation), and reproducibility as the standard deviation of these medians. The sign of the breath‐hold medians was retained so that accuracy conveyed direction and indicated whether a patient tends to hold their breath above or below the CT‐simulation reference. Because positive and negative deviations can cancel, this signed mean is not a magnitude measure of closeness to 0 and magnitude is conveyed instead by reproducibility and %WT. To calculate %WT, each sampled point during breath holds was labeled as within or outside our clinic's tolerance of ±3 mm. %WT was the number of points within tolerance divided by the total number of points across all breath holds (Figure 4). Longer breath holds add more samples to the %WT and therefore carry more weight for the patient.

Once accuracy, reproducibility, and %WT were calculated, patients were stratified based on previously described clinical criteria (e.g., age, pain). Statistical comparisons of breath‐hold performance between patient groups were performed using the Mann–Whitney *U* test, with statistical significance set at *p* < 0.05.

## 3 RESULTS

**FIGURE 5:**
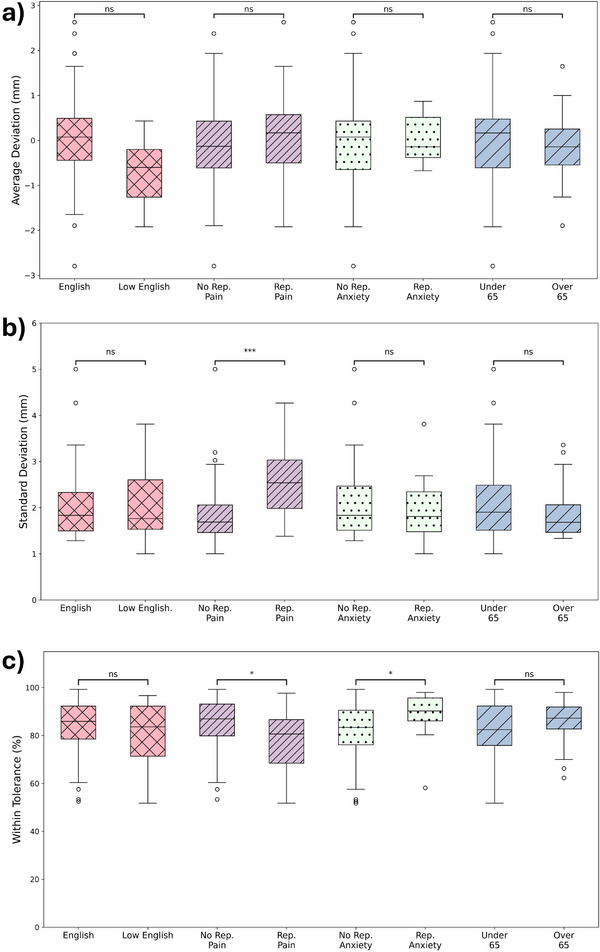
Box and whisker plots comparing groups of patients separated based on clinical characteristics for (a) breath‐hold accuracy (red cross hatch), (b) reproducibility (purple dense diagonal lines), and (c) percentage of time within tolerance (blue sparse diagonal lines). *p < 0.05, **p < 0.01, ***p < 0.001, ns = non‐significant.

Figure 5 shows box and whisker plots comparing accuracy (a), reproducibility (b), and %WT (c) for the patient groups analyzed in this study.

### 3.1 Breath‐hold accuracy

Mann–Whitney *U* tests comparing the median vertical deltas between patient groups indicated no statistically significant differences in accuracy. The largest observed difference in accuracy was between patients grouped by English proficiency. Patients with high English proficiency demonstrated a median accuracy of 0.07 ± 0.5 mm, whereas patients with low English proficiency exhibited a median accuracy of −0.60 ± 0.5 mm. Although not statistically significant (*p* = 0.4), this represented the most notable variation in accuracy among the groups analyzed (Figure 5).

Across the entire patient cohort, the accuracy was −0.08 mm, reflecting this patient population's ability to maintain accuracy during breath holds during DIBH treatment.

### 3.2 Breath‐hold reproducibility

The overall standard deviation in breath‐hold amplitude for all patients was 1.83 ± 0.42 mm. Among clinical factors examined, patient‐reported pain was the only characteristic significantly associated with breath‐hold reproducibility. Patients experiencing pain (≥1 on a 10‐point scale) exhibited significantly poorer reproducibility, with a standard deviation of 2.54 ± 0.51 mm compared to 1.69 ± 0.29 mm in patients without pain (*p* = 0.0003). This suggests that pain interferes with patients’ ability to consistently maintain stable breath holds.

### 3.3 Percentage of time within tolerance

The median percentage of time within tolerance across all patients was 85.9 ± 6.9%. Patients reporting anxiety had significantly lower %WT compared to those without anxiety (83.3 ± 10.46% vs. 90.2 ± 4.79%, *p* = 0.03). Similarly, patients experiencing pain demonstrated a lower %WT than pain‐free patients (80.6 ± 9.1% vs. 86.9 ± 6.6%, *p* = 0.02). These findings align with breath‐hold reproducibility results, indicating that physical and psychological discomfort negatively affect patients’ breath‐hold consistency.

## 4 DISCUSSION

This study examined how clinical factors, including English proficiency, patient‐reported pain and anxiety, and age, affect breath‐hold performance during DIBH breast cancer treatments. Smoking status was not included in the analysis due to the limited number of active smokers (*n* = 1) and variable smoking histories, which prevented meaningful subgroup comparisons.

Breath‐hold performance was evaluated using three metrics: accuracy (relative to the reference surface used for planning), reproducibility (independent of breath‐hold accuracy), and the percentage of time within tolerance. Using unweighted, per‐breath‐hold accuracy and reproducibility prevented a few prolonged breath holds from dominating a patient's performance, while %WT provides a duration‐sensitive view. Considered together, these metrics reduce the risk that patients with many short breath holds are systematically favored or penalized. All breath holds performed during setup, imaging, and treatment were analyzed for their impact on clinical workflow.

Overall breath‐hold performance in our patient population appears similar to others published. The reproducibility in breath holds across the population analyzed was 1.83 mm, which is similar to the 1.0–1.2 mm reproducibility reported by Huijskens et al.^17^ who also analyzed a set of 33 left‐sided breast cancer patients treated with SGRT and monitored with AlignRT. For breath‐hold accuracy, the mean positional error reported by Kalet et al.,^18^ which analyzed 64 left‐sided breast cancer patients monitored with Calypso or C‐RAD, was 0.33 mm and we observed a mean positional error of −0.08 mm in this work.

Our analysis found no significant association between patient age and breath‐hold performance, indicating that age alone should not exclude patients from DIBH. English proficiency similarly showed no statistically significant differences, also suggesting that the need for a translator should not prevent patients from receiving DIBH treatments. However, patients with low English proficiency consistently demonstrated reduced breath‐hold amplitudes during treatment compared to CT simulation. This may be due to limited real‐time guidance for non‐English‐speaking patients, who typically only receive initial instructions in their native language in contrast to the more corrective cues (e.g., “breathe in a little more”) provided to English‐speaking patients. These findings potentially highlight the value of developing targeted educational materials or resources to support patients with limited English proficiency. Although the difference in median accuracy (0.07 mm for high proficiency vs. −0.60 mm for low proficiency, *p* = 0.4) was not statistically significant, the small size of the low proficiency group (*n* = 8 of 72) may have reduced the statistical power of our analysis. Additional studies with larger sample sizes are needed to more definitively assess the impact of language proficiency on breath‐hold performance.

Conversely, patient‐reported pain and anxiety were significantly associated with impaired breath‐hold performance. Patients who reported pain during consultation exhibited reduced reproducibility (standard deviation: 2.54 mm vs. 1.69 mm, *p* = 0.0003) and a lower percentage of breath holds within tolerance (80.6% vs. 86.9%, *p* = 0.02). Similarly, reported anxiety was associated with a notable reduction in %WT (83.3% vs. 90.2%, *p* = 0.03), suggesting that psychological stress may also hinder a patient's ability to maintain consistent breath holds. Clinically, these associations may lengthen treatments through repeated or interrupted breath holds and more frequent coaching. Although we did not evaluate treatment duration in this work, a meaningful analysis would require separation of setup and imaging breath‐holds from delivery breath‐holds.

These findings are consistent with qualitative results from Dower et al.,^19^ who surveyed 22 DIBH breast cancer patients and found that fear and anxiety often interfered with a patient's confidence in their ability to perform breath holds consistently. Patients also described pain associated with maintaining the breath‐hold position, particularly due to prolonged arm elevations, which further exacerbated discomfort.^19^

Several institutions, including Memorial Sloan Kettering Cancer Center^20^ and the American Cancer Society,^21^ recommend that patients take either over the counter or prescribed pain medications prior to radiation therapy sessions. This practice is particularly advised for procedures where patients must remain still for extended periods of time, such as DIBH breast treatments. In addition to pharmacological interventions, non‐medicinal approaches have shown promise in managing cancer‐related pain. Techniques such as relaxation exercises and mindfulness‐based stress reduction have also been shown to serve as complementary therapies to traditional pain management strategies.^22^

Together, these results suggest that both emotional and physical distress can meaningfully compromise breath‐hold performance. Addressing these issues through improved patient education, physical support measures (such as arm positioning aids), and anxiety‐reducing interventions (either medicinal or psychological) may help improve treatment compliance and dosimetric outcomes.

Educational initiatives have also been proven effective in improving breath‐hold outcomes. Kim et al.^23^ reported that preparatory coaching sessions prior to CT simulation improved both breath‐hold accuracy and reproducibility. Oonsiri et al.^24^ also found that the use of educational materials not only reduced patient stress but also shortened simulation time, improving efficiency for both patients and staff. Tailoring educational materials to accommodate language barriers, such as offering translations, may be particularly beneficial for patients with limited English proficiency.

Given the significant cardiac‐sparing benefits of DIBH for patients with left‐sided breast cancer, no patients should be excluded from this treatment based on potential breath‐hold difficulties. Instead, our findings suggest that patients experiencing physiological or psychological distress could benefit from pre‐treatment interventions such as pain management, additional supportive devices, or anxiety reduction strategies. Rather than serving as exclusion criteria, these results should guide the development of mitigation strategies to ensure that all eligible patients can successfully undergo DIBH and receive its associated heart dose reduction benefits.

## 5 CONCLUSIONS

This study evaluated how clinical factors influence breath‐hold performance during DIBH for patients with left‐sided breast cancer. While patient age and English proficiency were not significantly associated with performance differences, pain and anxiety were linked to worse reproducibility and reduced time within tolerance. These findings suggest that physical discomfort and psychological stress can compromise treatment consistency and should be proactively addressed.

Importantly, factors like pain and anxiety should not be viewed as contraindications for DIBH, given the significant heart dose‐sparing benefits it provides. Instead, they present opportunities for supportive intervention (such as pain management, anxiety‐reducing strategies, coaching, and translated educational resources) to improve patient experience and treatment outcomes.

While this was a single‐institution study with AlignRT, we expect the findings in this work to generalize beyond our specific setup. The observed effects are driven by patient‐level factors, with pain and anxiety associated with less stable breath holds, so the direction of the results should hold across SGRT vendors and DIBH workflows. Absolute values, particularly %WT, may vary with implementation details such as clinic‐specific thresholds, ROI selection, and software version. Results may also differ when using techniques such as ABC or internal surrogates.

By identifying patients who may benefit from targeted interventions in advance, clinics can enhance the effectiveness of DIBH, minimize treatment delays, and ensure equitable access to its clinical benefits. These results can further inform future development of patient selection guidelines and supportive care protocols for breast radiation therapy. This was a retrospective, single‐institution study, and some patient groups had limited numbers, which reduces precision and broad generalizability. Even so, the consistent associations with pain and anxiety suggest that addressing these factors can improve DIBH stability and treatment efficiency.

## AUTHOR CONTRIBUTIONS

Kaelyn Becker performed the roles of data curation, formal analysis, validation, writing—original draft, and writing—review and editing. Kelly Kisling performed the roles of conceptualization, methodology, project administration, and writing—review and editing. Laura Padilla performed the roles of conceptualization, methodology, project administration, and writing—review and editing.

## CONFLICT OF INTEREST STATEMENT

Kelly Kisling has received speaker fees from VisionRT. Laura Padilla has a research agreement with VisionRT.

## References

[1] Early Breast Cancer Trialists’ Collaborative Group . Effects of radiotherapy and of differences in the extent of surgery for early breast cancer on local recurrence and 15‐year survival: an overview of the randomised trials. Lancet. 2005;366(9503):2087‐2106. doi:10.1016/S0140‐6736(05)67887‐7 16360786 10.1016/S0140-6736(05)67887-7

[2] Cuzick J , Stewart HJ , Peto R . Overview of randomized trials of postoperative adjuvant radiotherapy in breast cancer. Cancer Treat Rep. 1987;71(1):15. 2856861

[3] Fisher B , Anderson S , Bryant J , et al. Twenty‐year follow‐up of a randomized trial comparing total mastectomy, lumpectomy, and lumpectomy plus irradiation for the treatment of invasive breast cancer. N Engl J Med. 2002;347(16):1233‐1241. doi:10.1056/NEJMoa022152 12393820 10.1056/NEJMoa022152

[4] Lundstedt D , Gustafsson M , Steineck G , et al. Risk factors of developing long‐lasting breast pain after breast cancer radiotherapy. Int J Radiat Oncol Biol Phys. 2012;83(1):71‐78. doi:10.1016/j.ijrobp.2011.05.065 22079722 10.1016/j.ijrobp.2011.05.065

[5] Taylor C , Correa C , Duane FK , et al. Estimating the risks of breast cancer radiotherapy: evidence from modern radiation doses to the lungs and heart and from previous randomized trials. J Clin Oncol. 2017;35(15):1641‐1649. doi:10.1200/JCO.2016.72.0722 28319436 10.1200/JCO.2016.72.0722 PMC5548226

[6] Pierce SM , Recht A , Lingos TI , et al. Long‐term radiation complications following conservative surgery (CS) and radiation therapy (RT) in patients with early stage breast cancer. Int J Radiat Oncol Biol Phys. 1992;23(5):915‐923. doi:10.1016/0360‐3016(92)90895‐O 1639653 10.1016/0360-3016(92)90895-o

[7] Rutqvist LE , Johansson H . Mortality by laterality of the primary tumour among 55,000 breast cancer patients from the Swedish Cancer Registry. Br J Cancer. 1990;61:866‐868. doi:10.1038/bjc.1990.193 2372488 10.1038/bjc.1990.193 PMC1971705

[8] Darby SC , Ewertz M , McGale P , et al. Risk of ischemic heart disease in women after radiotherapy for breast cancer. New Eng J Med. 2013;368(11):987‐998. doi:10.1056/NEJMoa1209825 23484825 10.1056/NEJMoa1209825

[9] Misra S , Mishra A , Lal P , et al. Cardiac dose reduction using deep inspiratory breath hold (DIBH) in radiation treatment of left sided breast cancer patients with breast conservation surgery and modified radical mastectomy. J Med Imaging Radiat Sci. 2021;52(1):57‐67. doi:10.1016/j.jmir.2020.12.004 33509700 10.1016/j.jmir.2020.12.004

[10] Smyth LM , Knight KA , Aarons YK , Wasiak J . The cardiac dose‐sparing benefits of deep inspiration breath‐hold in left breast irradiation: a systematic review. J Med Radiat Sci. 2015;62(1):66‐73. doi:10.1002/jmrs.89 26229669 10.1002/jmrs.89 PMC4364808

[11] Corradini S , Angelini L , Gaasch A , et al. Final results from the SAVE‐HEART study—surface‐based deep inspiration breath‐hold radiotherapy in left‐sided breast cancer. Int J Radiat Oncol Biol Phys. 2024;120:304‐305. doi:10.1016/j.ijrobp.2024.07.675 10.1016/j.esmoop.2024.103993 PMC11655685 39631360

[12] Mittauer KE , Deraniyagala R , Li JG , et al. Monitoring ABC‐assisted deep inspiration breath hold for left‐sided breast radiotherapy with an optical tracking system. Med Phys. 2015;42(1):134‐143. doi:10.1118/1.4903511 25563254 10.1118/1.4903511

[13] Penninkhof J , Fremeijer K , Offereins‐van Harten K , et al. Evaluation of image‐guided and surface‐guided radiotherapy for breast cancer patients treated in deep inspiration breath‐hold: a single institution experience. Tech Innov Patient Support Radiat Oncol. 2022;21:51‐57. doi:10.1016/j.tipsro.2022.02.001 35243045 10.1016/j.tipsro.2022.02.001 PMC8861395

[14] Betgen A , Alderliesten T , Sonke J , van Vliet‐Vroegindeweij C , Bartelink H , Remeijer P . Assessment of set‐up variability during deep inspiration breath hold radiotherapy for breast cancer patients by 3D‐surface imaging. Radiother Oncol. 2013;106(2):225‐230. doi:10.1016/j.radonc.2012.12.016 23414819 10.1016/j.radonc.2012.12.016

[15] Zhao H , Sarkar V , Paxton A , et al. Clinical evaluation of a newly released surface‐guided radiation therapy system on DIBH for left breast radiation therapy. Med Phys. 2023;50(10):5978‐5986. doi:10.1002/mp.16699 37683108 10.1002/mp.16699

[16] Laaksomaa M , Moser T , Kritz J , Pynnönen K , Rossi M . Comparison of three differently shaped ROIs in free breathing breast radiotherapy setup using surface guidance with AlignRT. Rep Pract Oncol Radiother. 2021;26:545‐552. 34434570 10.5603/RPOR.a2021.0062 PMC8382080

[17] Huijskens S , Granton P , Fremeijer K , et al. Clinical practicality and patient performance for surface‐guided automated VMAT gating for DIBH breast cancer radiotherapy. Radiother Oncol. 2024;195:110229. doi:10.1016/j.radonc.2024.110229 38492672 10.1016/j.radonc.2024.110229

[18] Kalet AM , Cao N , Smith WP , et al. Accuracy and stability of deep inspiration breath hold in gated breast radiotherapy–a comparison of two tracking and guidance systems. Phys Med. 2019;60:174‐181. doi:10.1016/j.ejmp.2019.03.025 31000080 10.1016/j.ejmp.2019.03.025

[19] Dower K , Halkett GK , Dhillon H , Naehrig D , O'Connor M . Eliciting the views of left breast cancer patients’ receiving deep inspiration breath hold radiation therapy to inform the design of multimedia education and improve patient‐centered care for prospective patients. J Med Radiat Sci. 2024;71:384‐395. doi:10.1002/jmrs.790 38623813 10.1002/jmrs.790 PMC11569405

[20] Memorial Sloan Kettering Cancer Center . Radiation therapy to your breast or chest wall. 2023. Accessed May 12, 2025. https://www.mskcc.org/cancer‐care/patient‐education/radiation‐therapy‐breast‐chest‐wall

[21] American Cancer Society . Opioids for cancer pain. 2024. Accessed May 12, 2025. https://www.cancer.org/cancer/managing‐cancer/side‐effects/pain/cancer‐pain/opioid‐pain‐medicines‐for‐cancer‐pain.html

[22] Mayr NA , Borm KJ , Kalet AM , et al. Reducing cardiac radiation dose from breast cancer radiation therapy with breath hold training and cognitive behavioral therapy. Top Magn Reson Imaging. 2020;29:135‐148. doi:10.1097/RMR.0000000000000241 32568976 10.1097/RMR.0000000000000241

[23] Kim A , Kalet A , Cao N , et al. Effects of preparatory coaching and home practice for deep inspiration breath hold on cardiac dose for left breast radiation therapy. Clin Oncol. 2018;30:571‐577. doi:10.1016/j.clon.2018.04.009 10.1016/j.clon.2018.04.009 29773446

[24] Oonsiri P , Wisetrinthong M , Chitnok M , Saksornchai K , Suriyapee S . An effective patient training for deep inspiration breath hold technique of left‐sided breast on computed tomography simulation procedure at King Chulalongkorn Memorial Hospital. Radiat Oncol J. 2019;37:201. doi:10.3857/roj.2019.00290 31591868 10.3857/roj.2019.00290 PMC6790791

